# Novel Aptamer-Nanoparticle Bioconjugates Enhances Delivery of Anticancer Drug to MUC1-Positive Cancer Cells *In Vitro*

**DOI:** 10.1371/journal.pone.0024077

**Published:** 2011-09-01

**Authors:** Chenchen Yu, Yan Hu, Jinhong Duan, Wei Yuan, Chen Wang, Haiyan Xu, Xian-Da Yang

**Affiliations:** 1 Institute of Basic Medical Sciences, Chinese Academy of Medical Sciences & Peking Union Medical College, Beijing, China; 2 State Key Laboratory of Molecular Oncology, Cancer Institute & Hospital, Chinese Academy of Medical Sciences, Peking Union Medical College, Beijing, China; 3 National Center of Nanoscience and Technology, Beijing, China; University of Queensland, Australia

## Abstract

MUC1 protein is an attractive target for anticancer drug delivery owing to its overexpression in most adenocarcinomas. In this study, a reported MUC1 protein aptamer is exploited as the targeting agent of a nanoparticle-based drug delivery system. Paclitaxel (PTX) loaded poly (lactic-co-glycolic-acid) (PLGA) nanoparticles were formulated by an emulsion/evaporation method, and MUC1 aptamers (Apt) were conjugated to the particle surface through a DNA spacer. The aptamer conjugated nanoparticles (Apt-NPs) are about 225.3 nm in size with a stable *in vitro* drug release profile. Using MCF-7 breast cancer cell as a MUC1-overexpressing model, the MUC1 aptamer increased the uptake of nanoparticles into the target cells as measured by flow cytometry. Moreover, the PTX loaded Apt-NPs enhanced *in vitro* drug delivery and cytotoxicity to MUC1^+^ cancer cells, as compared with non-targeted nanoparticles that lack the MUC1 aptamer (P<0.01). The behavior of this novel aptamer-nanoparticle bioconjugates suggests that MUC1 aptamers may have application potential in targeted drug delivery towards MUC1-overexpressing tumors.

## Introduction

Chemotherapy is a primary treatment for cancer, but its efficacy is often limited due to associated adverse effects. Targeted drug delivery system may overcome the non-specific toxicity of chemotherapy because it may direct anticancer drugs to tumor cells and avoid the toxicity to normal cells. In many cases, nanoparticle (NP) has been proved of great potential for drug delivery due to the passive tumor-targeting effect of enhanced permeability and retention (EPR) exhibited by most nano-carriers [1]. Moreover, an active targeting effect could be realized if a targeting agent is conjugated to nanoparticle surface. In the past, monoclonal antibodies (mABs) were the proposed targeting agents [2]. Lately, novel targeting agents, including aptamers [3], short peptides [4] and other small molecules [5], have become the new generation targeting molecules. Aptamers are small strands of DNA or RNA that could form unique 3-dimensional structures that specifically combine to molecular targets with high affinity. Comparing to other targeting agents, aptamers possess distinctive advantages: low synthesis cost, low-immunogenicity, small size that makes it easy to penetrate through solid tumors, and high affinity comparable to monoclonal antibodies for binding to almost any molecules. Previously, aptamers have been successfully applied as targeting agents to enhance drug delivery to prostate cancer [6], [7], [8] and lymphoblastic leukemia cells [9].

The above studies have paved the road for utilizing aptamer-conjugated nanoparticle to develop targeted drug delivery systems. However, most aptamer-guided nanoparticles studied so far aimed at prostate cancer [6], [7], [8], and aptamer-guided nano-carriers for drug delivery to other cancers have not been reported in literature. It would be preferable if an aptamer targeting a broad spectrum of cancers is employed to construct a targeted drug delivery system. MUC1 mucin is a large transmembrane glycoprotein, whose expression increased at least 10-fold in most malignant adenocarcinomas, making it an ideal target molecule for chemotherapeutics [10]. The glycosylation and distribution of the protein at the cell surface are abnormal in ovarian, lung, pancreatic and prostate cancers, as well as in primary and metastatic breast cancers, which is the most common cancer in women with 1.4 million cases reported in 2008 [11]. Recently, several MUC1 aptamers were developed by C.S.M. Ferreira et al. [12], and one of them was applied to selectively deliver phototoxin to cancer cells *in vitro* [13]. So far, however, nanoparticle-based anticancer drug delivery system targeting the MUC1 protein has not been reported in literature. Among the published MUC1 aptamers, S2.2 is a 25-base oligonucleotide that binds to MUC1 protein with relatively high affinity and specificity [12], [13]. In this study, we constructed a MUC1 aptamer-conjugated nanoparticle with S2.2 for delivery of paclitaxel to MUC1-positive tumor cells, combining the advantages of aptamer as targeting agent and the strengths of nanoparticle as drug carrier. We examined its basic properties and evaluated its delivery efficacy *in vitro* using a MUC1-overexpressing model MCF-7 cell line. We now report that the MUC1 aptamer-nanoparticle enhances the delivery of anticancer drug to MUC1-positive MCF-7 cells *in vitro*.

## Materials and Methods

### Materials

MUC1 aptamer S2.2 (5′-GCA GTT GAT CCT TTG GAT ACC CTG G-3′) was synthesized by Invitrogen (Shanghai, China). A modified aptamer S2.2-spacer, made of S2.2 and a designed DNA spacer (5′-GCA GTT GAT CCT TTG GAT ACC CTG GTT CCC TTC CTT CTC TCT TCC TCT CTC CTT CTC TCT TCC TCT CTC CTT C-3′) was also synthesized. Some aptamers were modified with 3′-NH_2_ or 5′-FITC on an as needed base. Poly (lactic-co-glycolic-acid) (PLGA, 50∶50, MW = 16,000), *N*-hydroxysulfosuccinimide (NHS), 1-ethyl-3-(3-dimethylaminopropyl) carbodiimide hydrochloride (EDC), paclitaxel (PTX), 4′,6-diamidino-2-phenylindole (DAPI), fluorescein isothiocyanate (FITC) and poloxamer 188 were all of analytical grade.

MCF-7 and HepG2 cell lines were obtained from the Cell Center of Chinese Academy of Medical Sciences (Beijing, China). Cells were cultured in DMEM medium, supplemented with 100 units/ml aqueous penicillin G, 100 mg/mL streptomycin, and 10% FBS at concentrations to allow 70% confluence in 24 h.

### Nanoparticle preparation

The paclitaxel encapsulated nanoparticles were prepared using an emulsion/evaporation technique. Briefly, 0.1 mg PTX and 2.0 mg PLGA was dissolved in 0.2 ml ethyl acetate and mixed with 1.0 ml 5% poloxamer aqueous solution for 30 s with sonication (200 w) in an ice bath. The emulsion was gently stirred and solvent evaporated at 40°C for 2 h. After evaporation, the suspension was centrifuged at 100,000 g for 30 min. The supernatant was removed and the precipitation was resuspended in double-distilled water. For evaluation of the cellular uptake of Apt-NPs, FITC was encapsulated into nanoparticles instead of PTX, at the concentration of 0.25 mg/ml.

### Characterization of nanoparticles

The particle size was determined by dynamic light scattering (Malvern Zetasizer Nano ZS, UK). 1.0 mg NPs or Apt-NPs were dissolved in 1.0 ml double-distilled water. The particle size distributions were measured at a scattering angle of 90°. The intensity-weighted mean value was recorded as the average of three measurements.

To determine the PTX loading, 1.0 mg lyophilized PTX-Apt-NPs were lysed in NaOH (1 M) and the UV absorbance (Thermo Nanodrop 1000, US) was measured at a wavelength of 227 nm. 1.0 mg lyophilized plain NPs were used as parallel controls to eliminate the impacts of the background. The PTX was quantitatively determined by comparing to a standard curve.

### Conjugation of aptamers to nanoparticles

The conjugation of aptamers or random DNA to nanoparticles was accomplished via crosslinking of –COOH and –NH_2_. Briefly, 50 µl of PTX-NPs (10 µg/ml in DNase RNase-free water) was incubated with 100 µl of 40 mM 1-ethyl-3-(3-dimethylaminopropyl) carbodiimide hydrochloride (EDC) and 100 µl of 10 mM *N*-hydroxysulfosuccinimide (NHS) for 15 minutes at room temperature with gentle stirring. Then the activated particles were covalently linked to 50 µl of 3′-NH_2_–modified MUC1 aptamer (1 µg/ml in DNase RNase-free water) or 3′-NH_2_, 5′-FITC–modified MUC1 aptamer on an as needed base, for 2 hours at room temperature. The resulting aptamer-nanoparticle bioconjugates were washed, resuspended, and preserved in DNase RNase-free water. The conjugation of 5′-FITC, 3′-NH_2_–modified MUC1 aptamer to PLGA microparticles (0.5 mg/ml) was analyzed by the flow cytometry (Accuri C6, US).

### Cellular binding of aptamers

The cellular binding of aptamers was determined by flow cytometry (FCM) analysis of cells after incubation with FITC-labeled random DNA (Ran), S2.2 aptamer, or S2.2-spacer. MCF-7 and HepG2 cells were gently scraped and washed with D Hank's twice. The cells were suspended in the binding buffer (100 mM NaCl, 5 mM MgCl_2_, pH 7.2) and incubated with Ran, MUC1 aptamer S2.2 or S2.2-spacer at the concentration of 300 nM for 30 min. The FCM analysis was performed to examine the binding of random DNA or aptamers to both cell lines.

### *In vitro* release profile of PTX from Apt-NPs

The *in vitro* release of PTX from Apt-NPs was detected by the membrane diffusion technique. PTX-Apt-NPs were suspended in phosphate buffer saline (PBS, pH 7.4) containing 0.5% (w/v) poloxamer. 5 ml of the suspension (2 mg/ml) was introduced into a dialysis bag (MWCO 3500) and then immersed into 95 ml release medium in an incubator shaker set at 120 rpm at 37°C. At the predetermined time intervals, samples were withdrawn and replaced with fresh release medium. Nanoparticles and released PTX were separated by ultra-centrifugation at 100,000 g for 30 min at 4°C. The PTX content in the supernatant was determined by UV spectrophotometer at a wavelength of 227 nm. The cumulative release percentages of PTX from Apt-NPs were calculated as follows and plotted against time.
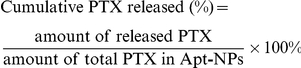



### Cellular uptake experiment

The cellular uptake of the particles was determined by FCM analysis of cells after incubation with FITC encapsulated particles. MCF-7 and HepG2 cells were grown in 24-well plates for 24 h. The cells were then incubated with 50 µg/ml FITC encapsulated NPs or Apt-NPs for 2 h at 37°C. The FCM analysis was then performed to examine the cellular fluorescence uptake of both cancer cell lines.

### Confocal fluorescence scanning microscopy

The cellular uptake of NPs or Apt-NPs by MCF-7 was further studied by confocal fluorescence scanning microscopy (Perkin Elmer Ultraview, US). MCF-7 cells were allowed to adhere to a glass cover slip in 6-well plate for 24 h. The cells were then incubated with 100 µg/ml NPs or Apt-NPs, both containing FITC, for 2 h at 37°C. Then the cells were washed with D Hank's and treated with 0.25% trypsin for 1 min. The suspended cells were transferred to a centrifuge tube and washed twice. The cells were then fixed with 4% formaldehyde for 10 min at 4°C and analyzed by confocal fluorescence scanning microscopy.

### *In vitro* cytotoxicity

To evaluate the cytotoxicity effects of PTX-Apt-NPs against MCF-7 and HepG2 cells, both cell lines were grown in 96-well plates. The cells were treated with plain NPs, free PTX, PTX-loaded NPs (PTX-NPs) and PTX-loaded Apt-NPs (PTX-Apt-NPs). PTX-loaded NPs conjugated to random DNA (PTX-R-NPs) was also used to serve as another control. MCF-7 and HepG2 cells were co-cultured with the respective substances at an equivalent PTX dose of 0.05 µg/ml for 4 h at 37°C, then washed with D Hank's (2×500 µl per well). Cells were cultured for a further 48 h and then MTS assay (Promega, US) was used to determine the cell viability per standard protocol outlined by the manufacture.

### Statistical analysis

Statistical analysis was performed using Statistical Analysis System (SAS, Version 9.2). One-way ANOVA with Fisher's least significant difference (LSD) post hoc comparisons at 99% confidence interval was used for statistical comparisons. All data are presented as a mean value with its standard deviation indicated (mean ± SD).

## Results

### Preparation of Apt-NPs

**Figure 1:**
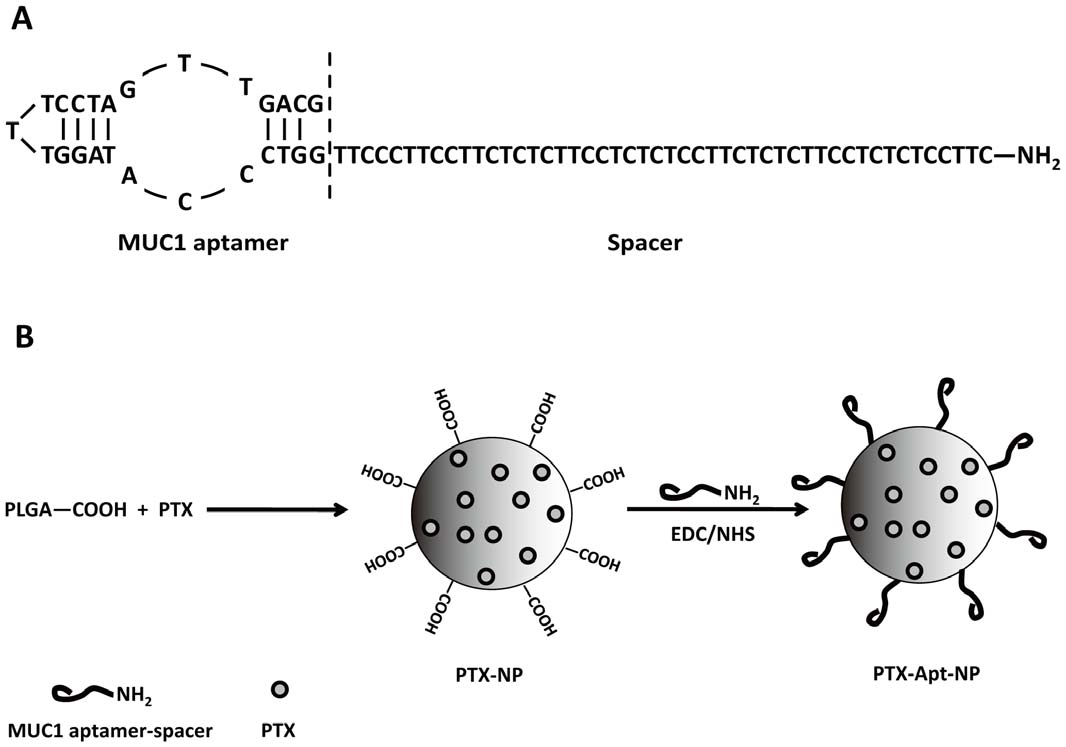
Construction of the S2.2-spacer aptamer and PTX-Apt-NPs. (A) Structure of MUC1 aptamer S2.2-spacer. The S2.2-spacer is made of the MUC1 aptamer S2.2 (as the targeting agent) and a designed sequence of single strand DNA (as the linking spacer). (B) Preparation procedure for PTX-Apt-NP using the emulsion/evaporation method.

PTX-Apt-NPs were constructed as outlined in Fig. 1 using a routine emulsion/evaporation method. PTX was incorporated into NPs by physical entrapment through hydrophobic interactions between PTX and PLGA (Fig. 1B). In order to facilitate polyvalent binding between the aptamer and the target, a spacer is often inserted between the aptamer and the nanoparticle vehicle. Hence we constructed a DNA spacer by extending the 3′ end of the original S2.2 aptamer with 48 additional bases (Fig. 1A), providing a spacer length close to that of PEG 3400, which is a commonly used spacer in targeted drug delivery systems [14], [15]. The extended portion of DNA will not affect the secondary structure of the S2.2 aptamer per computational analysis (RNAstructure, version 4.5). To conjugate the aptamer-spacer complex to PTX-loaded NPs, EDC and NHS were added to catalyze the formation of covalent couple between the -COOH of PLGA on the nanoparticle surface and the 3′-NH_2_ MUC1 aptamer (Fig. 1B). The drug loaded nanoparticle size is 164.0±7.6 nm before coupling to aptamers, and increased to 225.3±9.2 nm after the conjugation, presumably because of the added size of the aptamer and spacer. The PTX encapsulation efficacy was 83.6±1.7% with a drug load of 4.2±0.1%.

### Affinity of MUC1 aptamer to MCF-7 and HepG2 cell lines

**Figure 2:**
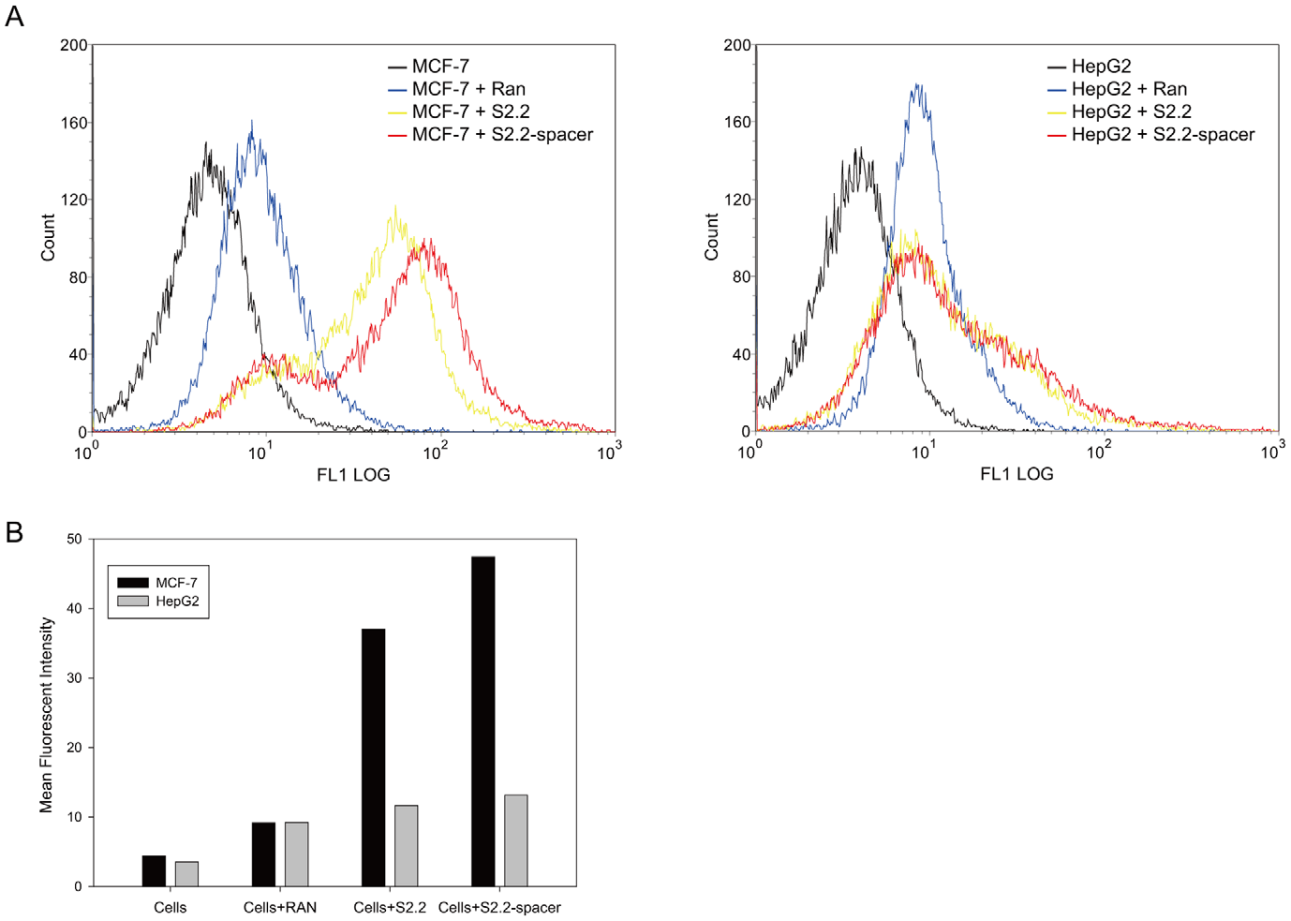
FCM analysis of MCF-7 and HepG2 cells incubated with random DNA, MUC1 aptamer S2.2, or S2.2-spacer. (A) Histograms of FCM analysis. FITC-labeled random DNA, S2.2, or S2.2-spacer was incubated separately with MCF-7 (left) and HepG2 (right) cells. (B) The mean fluorescent intensity of MCF-7 and HepG2 cells incubated with random DNA and different aptamers.

The aptamer S2.2 is reported to specifically bind to MUC1 protein with high affinity [12]. To test whether S2.2 would differentially bind to MUC1-positive and MUC1-negative cells, the binding of S2.2 to MCF-7 and HepG2 cells was evaluated by FCM analysis. A random DNA (Ran) was used as control. Previous study have well established that MCF-7 is a human breast cancer cell line that overexpresses MUC1 protein on its surface [16], and that HepG2 is a MUC1-negative human hepatic cancer cell line [17]. Consistent with the original report, the aptamer S2.2 (yellow curve) demonstrated a higher binding to the MUC1-positive MCF-7 cells than the MUC1-negative HepG2 cells (Fig. 2 A&B). The random DNA (blue curve) had a much lower binding to both MCF-7 and HepG2 cells, presumably resulted from non-specific binding. Furthermore, to examine if S2.2-spacer had a similar binding preference for the MUC1-positive cells, the binding experiment was repeated for S2.2-spacer. The results exhibited that the S2.2-spacer (red curve) had similar interaction with both cells as S2.2 (Fig. 2 A&B), suggesting that S2.2-spacer maintained the selective binding ability of the original aptamer. The mean fluorescent intensity of FITC-labeled S2.2-spacer binding with MCF-7 is 3.5 times higher than that with HepG2, making it a qualified targeting agent for selectively aiming at the MCF-7 cells. Since a spacer may facilitate polyvalent binding between the aptamer and the target, S2.2-spacer was applied as the targeting agent to construct the drug delivery system in this study.

### Evaluation of conjugation between aptamers and nanoparticles

**Figure 3:**
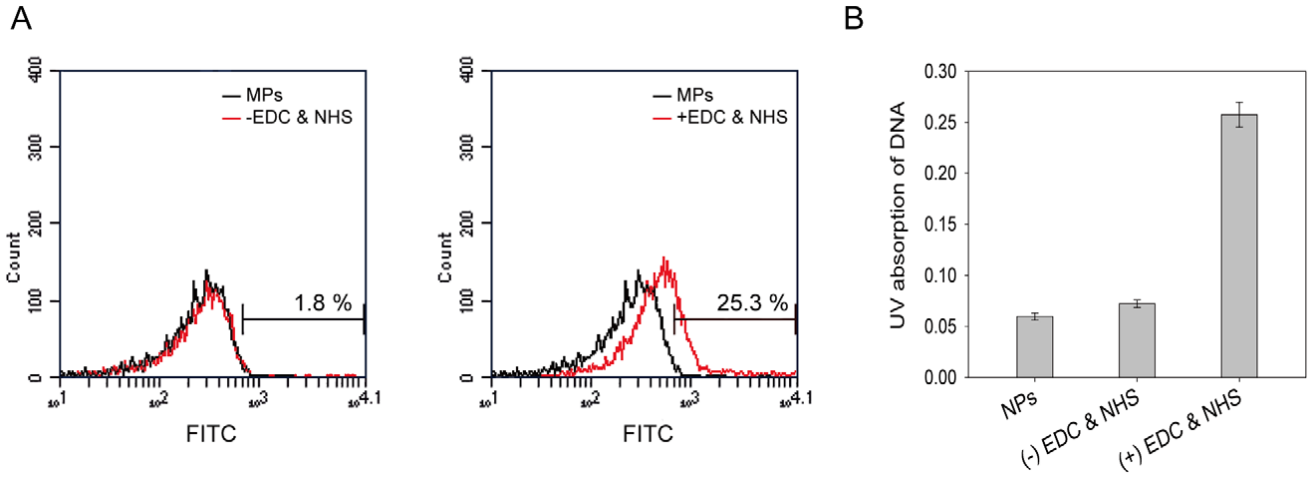
Analysis of aptamer conjugation to microparticles or nanoparticles. The PLGA microparticles (MPs) or nanoparticles (NPs) reacted with aptamers in the absence (-EDC&NHS) or presence (+EDC&NHS) of the catalysts. (A) Histograms of FCM analysis of MP reacted with FITC-labeled aptamers in the absence (-EDC&NHS, left) or presence (+EDC&NHS, right) of the catalysts. Plain microparticles that had not reacted with aptamers were used as control. (B) UV absorption of DNA at 260 nm (n = 3) on the particles that underwent conjugation process with aptamers. The control group (NPs) indicates plain nanoparticles that have not reacted with DNA aptamers.

The MUC1 aptamer-spacer was conjugated to nanoparticle in the reaction catalyzed by EDC and NHS. To ensure that the DNA aptamer was indeed linked to the surface of the particle, two experiments were conducted. In the first experiment, FITC-labeled aptamer was co-incubated with microparticles (MPs) either in the presence or in the absence of the catalysts EDC and NHS, and then analyzed by the FCM. As shown in Fig. 3A, the particle fluorescent intensity in the absence of EDC and NHS was nearly identical to that of the plain MPs, while a positive fluorescence was observed in the presence of EDC and NHS. The results suggested the conjugation between aptamers and particles was indispensably enabled by the catalysts, and that aptamers were stably conjugated to the particles through covalent linkage. The second experiment examined the presence of DNA aptamer on nanoparticles by UV spectroscopy and evaluated the conjugating efficacy. Similar to the FCM results, more aptamers were linked to nanoparticles in the presence of EDC and NHS (Fig. 3B). The amount of conjugated aptamers on NPs was also estimated. In this reaction system, approximately 0.1 nmol aptamers were covalently coupled to 1 mg PLGA NPs, or about 120 aptamers for each nanoparticle.

### *In vitro* release profile of PTX from Apt-NPs

**Figure 4:**
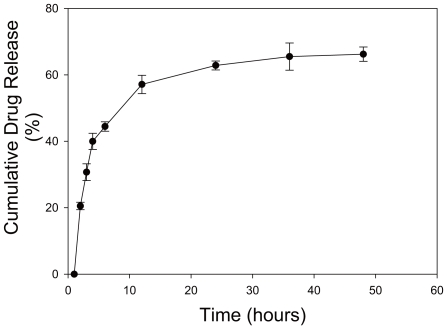
*In vitro* drug release profile of the PTX encapsulated Apt-NPs. The experiment was conducted in phosphate buffer saline (PBS, pH 7.4) containing 0.5% (w/v) poloxamer using the membrane diffusion technique, (n = 3).

We next studied the *in vitro* release profile of the PTX from the Apt-NPs, a necessary property for anticancer activity. The amount of PTX release in PBS was assayed overtime with UV absorption at 227 nm. A typical kinetics of sustained release process was observed for PTX, with about 65% of the drug gradually released over the first 48 hours (Fig. 4). The proportion of PTX released here was in line with the releasing profiles of many PLGA drug carrier systems reported in literature [18], [19], [20].

### Cellular uptake experiment

**Figure 5:**
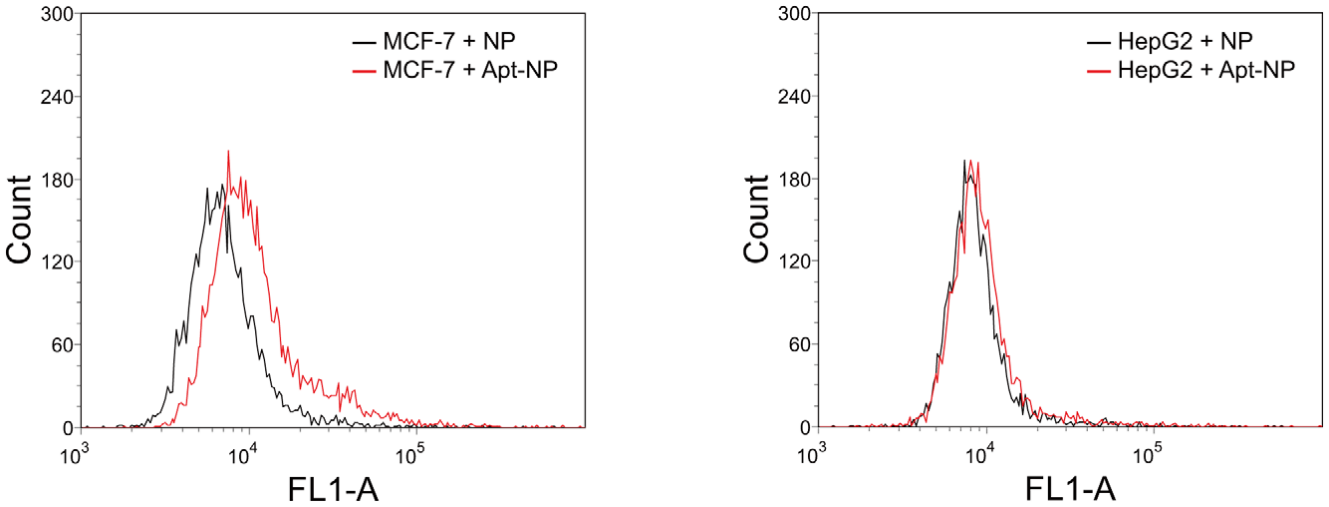
FCM analysis of cellular uptake of Apt-NPs and NPs by MCF-7 (left) and HepG2 cells (right). The cells were incubated with FITC encapsulated Apt-NPs or NPs at 50 µg/ml for 2 hours before subject to analysis.

The most important property of a targeted drug delivery system is its specificity towards the target cells. To explore the *in vitro* cancer targeting of the Apt-NPs against the MUC1-overexpressing cells, we compared the cellular uptake of MCF-7 (MUC1^+^) and HepG2 (MUC1^−^) using FCM analysis (Fig. 5). NPs and Apt-NPs, both encapsulating FITC, were co-cultured with MCF-7 and HepG2. Fluorescent intensity of MCF-7 and HepG2 cells incubated with NPs were similar in amplitude. However, for Apt-NPs treated MCF-7 cells, the fluorescent intensity increased by 71%, while that of HepG2 cells hardly changed. The distinction was presumably caused by the conjugated aptamers, which bound to the MUC1 protein on the surface of MCF-7 cells, resulting in more nanoparticles being attached to the cell surface and internalized into the cell.

### Confocal fluorescent scanning microscopy

**Figure 6:**
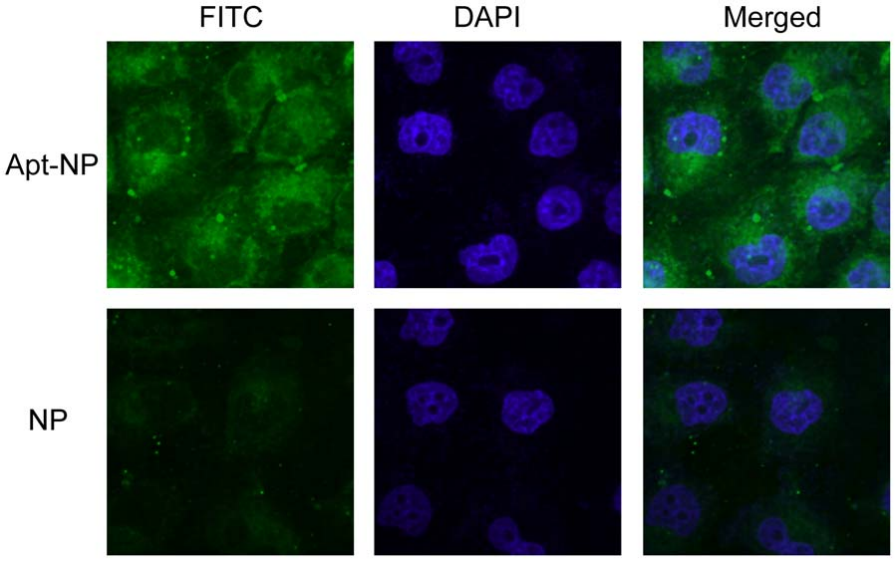
Confocal fluorescent scanning microscopy images detecting cellular uptake of Apt-NPs (top row) or NPs (bottom row) in MCF-7 cells. Green fluorescent FITC was encapsulated in Apt-NPs and NPs. The nuclei were stained blue with DAPI. The right column showed the merged images of the FITC and the DAPI channels. MCF-7 cells were exposed to FITC-encapsulated Apt-NPs or NPs at 100 µg/ml for 2 hours.

The above results showed that Apt-NPs generated enhanced fluorescence intensity in MUC1-positive MCF-7 cells, comparing with NPs. However, it was not entirely clear whether the Apt-NPs were attached to the cell surface or internalized into the cells. To further study the interaction scheme between Apt-NPs and the target cells, confocal fluorescent scanning microscopy was performed to determine the location of Apt-NPs. Multiple images scanning through various levels of the MCF-7 cells were obtained. The central level scans that went through the center of the cell and the nuclei were displayed in Fig. 6. The images clearly indicated that the Apt-NPs were mainly accumulated in the cytoplasm around the nucleus. Accordingly, Apt-NPs could be internalized into the target cells and carry the anticancer drugs into the cytoplasm. Compared to Apt-NPs, the amount of NPs entered MCF-7 cells was much less; suggesting again that the MUC1 aptamer facilitated the uptake of nanoparticles into the cells.

### *In vitro* cytotoxicity

**Figure 7:**
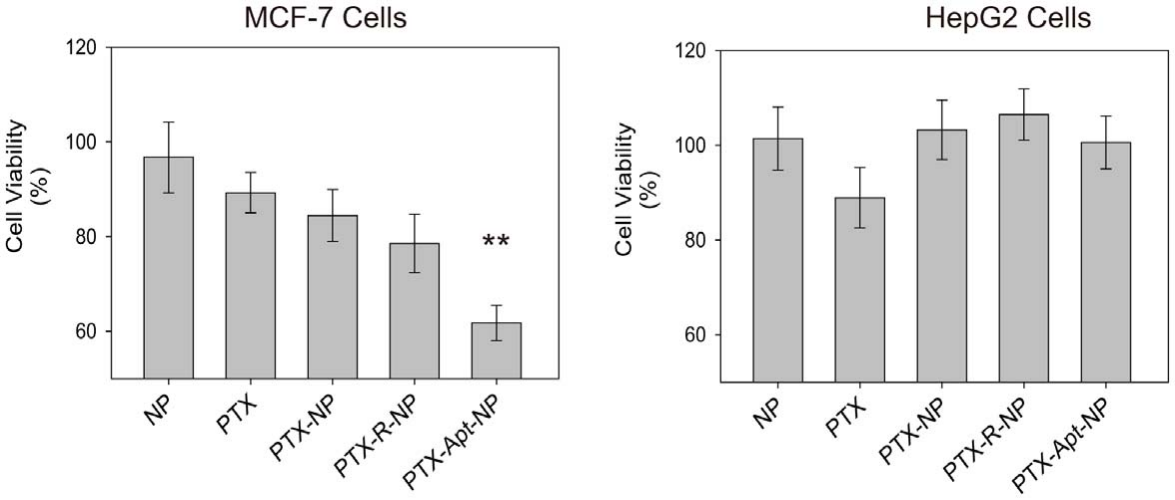
Cytotoxicity assays of MCF-7 (Left) and HepG2 cells (Right) treated for 4 hours with plain nanoparticles (NP), free paclitaxel (PTX), PTX-loaded NPs (PTX-NP), PTX-loaded NPs conjugated to random DNA (PTX-R-NP), or PTX-loaded NPs conjugated to MUC1 aptamer (PTX-Apt-NP). The cells were subsequently washed and incubated in culture media for a total of 48 hours, before cell viability in each group was assessed with a standard MTS assay (n = 6, mean ± SD).

The cellular uptake experiment showed that Apt-NPs increased the uptake of nanoparticles by MUC1-overexpressing tumor cells. However, it is unknown how this will affect the delivery of anticancer agent to the cells. To study the issue, *in vitro* cytotoxicity of free PTX, plain NPs, PTX-loaded NPs (PTX-NPs), PTX-loaded NPs conjugated to random DNA (PTX-R-NPs), and PTX-loaded NPs conjugated to MUC1 aptamers (PTX-Apt-NPs) were compared using MCF-7 (MUC1+) and HepG2 (MUC1-) as target cells. The results are presented in Fig. 7. Plain NPs showed little cytotoxicity, suggesting that the delivery vehicles are relatively nontoxic to the cells and that the cytotoxicity was mostly caused by the encapsulated PTX. Free PTX generated similar degrees of cytotoxicity in both cell lines. PTX-Apt-NPs produced a more potent cytotoxicity than PTX-NPs or PTX-R-NPs in MCF-7 cells (P<0.01). However, the cytotoxicity differences were not observed in HepG2 cells. This is consistent with the results shown in Fig. 5, in which MUC1 aptamer increased the uptake of Apt-NPs into MCF-7 cells (MUC1+) but not HepG2 cells (MUC1-). The results suggest that the MUC1 aptamer may selectively enhance the delivery of anticancer drug to MUC1-positive cancer cells.

## Discussion

Targeted drug delivery systems have been proposed to solve the problem that most anticancer therapeutic agents fail to act specifically on cancer cells and cause toxicity to normal cells. In this study, we designed a MUC1 aptamer-based targeted drug delivery system (Fig. 1) to enhance the delivery of paclitaxel to MUC1-overexpressing tumor cells. The MUC1 aptamers demonstrated higher affinity and selectivity against MCF-7 cells (MUC1^+^) over HepG2 cells (MUC1^−^) (Fig. 2). To construct the Apt-NPs, MUC1 aptamers were conjugated the aptamers to NPs surface through chemical covalent coupling (Fig. 3). The Apt-NPs showed a profile of sustained drug release (Fig. 4). The targeting aptamer increased the uptake of nanoparticles into MUC1-overexpressing tumor cells (Fig. 5 & 6). Moreover, the Apt-NPs enhanced the PTX delivery to the MUC1-positive tumor cells (Fig. 7) while showing no efficacy towards the control cells.

Previous studies of aptamer-conjugated NPs for drug delivery have improved efficacy against prostate cancer via an aptamer of prostate-specific membrane antigen (PSMA) [6], [7], [8], [21]. However, it would be ideal to construct an aptamer-based drug delivery system targeting a broad spectrum of cancers. MUC1 protein is overexpressed and aberrantly glycosylated on the surface of most adenocarcinoma cells [10], making it a very attractive target for cancer therapy. In this study, for the first time, MUC1 aptamer is exploited as a targeting agent in a nanoparticle-based drug delivery system. The MUC1 aptamer (S2.2) used in this study exhibited high binding affinity to MUC1, which is comparable to the anti-MUC1 antibody C595 [12], [22]. The targeted nanoparticle delivery system based on this MUC1 aptamer was evaluated for its targeting capability against the MCF-7 cells *in vitro*. Similar to the drug delivery system based on PSMA aptamer, we observed enhanced uptake of Apt-NPs by MUC1-overexpressing MCF-7 cells (Fig. 7). Since MUC1 protein is overexpressed on the surface of multiple types of cancer cells, such a drug delivery system may potentially improve the delivery of anticancer agents to multiple malignancies, such as pancreatic cancer [23], prostate cancer [24], ovarian cancer [25], etc.

As discussed above, the enhanced delivery of anticancer drug was mainly induced by the MUC1 aptamer. The confocal scanning microscopy images (Fig. 6) and the increased cytotoxicity comparing to free PTX (Fig. 7) indicated that the NPs were internalized into the cells. Generally it is thought that drug-loaded PLGA NPs are taken up by cells through internalization, and subsequently release the drug inside the cells [26]. The S2.2 aptamers conjugated to the NPs probably promoted the interaction between NPs and MCF-7 cells via ligand-receptor recognition. Specifically, the S2.2 bound to MUC1 presumably acted like an anchor and pulled the nanoparticle to the vicinity of the cell, and improved the chances of the nanoparticle being internalized by MCF-7 cells. For MUC1-negative HepG2 cells, the aptamer failed to enhance the uptake of nanoparticle (Fig. 5), presumably because it did not improve the interaction between the nanoparticle and the cell.

A spacer molecule is often needed between the targeting molecule and the carrier nanoparticle: the length and flexibility of the spacer allows the targeting aptamers penetrate through the cell surface molecules and bind to the targets in a polyvalent way [14]. In this study, we attempted a novel approach to construct a spacer, which could simplify the preparation procedure. We extended the 25-base S2.2 aptamer by adding a 48-base DNA sequence to the 3′ end of the aptamer, to make a 73-base strand. The DNA spacer was designed to avoid forming secondary structure and had approximately the same length as the mostly used spacer PEG-3400 (25 nm). The binding experiment (Fig. 2) proved that aptamer S2.2 and S2.2-spacer had similar binding profiles with the MUC1-positive target cells and MUC1-negative control cells. These results suggested that the extended DNA strand did not affect the binding capability of S2.2 and could serve as a feasible spacer. By avoiding the PEG spacer, this construction method simplified the chemistry of conjugating targeting agent and spacer to nanoparticle. Since the advantages of the spacer has been well approved [27], [28] and the S2.2-spacer demonstrated good affinity to MUC1-positive target cells, aptamers with DNA spacer were utilized to construct the targeted drug delivery system in this research. As expected, the S2.2-spacer conjugated Apt-NPs displayed high specificity and affinity towards MUC1-overexpressing cells *in vitro*.

MUC1 protein is an important tumor-associated antigen (TAA) detected in most adenocarcinomas. Recently, a group of MUC1 aptamers have been identified, including the aptamer S2.2 [12], which has the potential to serve as targeting agent for MUC1 protein. The primary goal of this study is to explore the possibility of developing a nanoscale drug delivery system based on S2.2 and to preliminarily evaluate its cancer-targeting capability *in vitro*. Here we constructed a novel PTX-loaded nanoparticle conjugated to MUC1 aptamer S2.2 through a DNA spacer. The aptamer was found to increase the uptake of nanoparticles into MUC1-positive MCF-7 cells. Moreover, the PTX-loaded Apt-NPs enhanced the cytotoxicity against MCF-7 cells *in vitro*. Nevertheless, in order to practically realize MUC1-targeted drug delivery, extensive future research on the Apt-NPs is still warranted, including detailed *in vivo* evaluation of pharmacokinetics, pharmacodynamics, adverse effects and long-term biocompatibility in animal studies.

### Conclusion

In summary, a MUC1 aptamer-guided nanoscale drug delivery system was developed with a novel conjugation strategy. The results demonstrate that the system can effectively enhance the PTX delivery to MUC1- overexpressing MCF-7 cells *in vitro*. Since many cancers overexpress MUC1 protein, we postulate that targeted drug delivery aiming at MUC1 may serve as a potential strategy to improve the treatment outcome of these tumors.
